# Enduring Cell Lines: Parents’ Experiences of Postmortem Tumor Banking in Childhood Cancer

**DOI:** 10.1177/10748407211001431

**Published:** 2021-04-15

**Authors:** Nancy J. Moules, Catherine M. Laing, Wendy Pelletier, Gregory M. T. Guilcher, Jennifer A. Chan

**Affiliations:** 1 University of Calgary, Alberta, Canada; 2 Alberta Children’s Hospital, Calgary, Canada

**Keywords:** pediatric oncology, childhood cancer, tumor banking, grief, hermeneutic research

## Abstract

While cure rates in pediatric oncology have improved over the past 30 years, childhood cancer remains the second leading cause of death in children aged 1 to 14. Developing therapies often require using cancerous tissues, which may come from deceased donors. Tumor banks collect, store, and distribute these donated samples. While tumor banking is more common, factors that contribute to parents’ decision and the impact of it on the family are not well understood. The purpose of this hermeneutic study was to understand the meaning and impact of tumor banking for parents of children who have died from cancer. Findings suggest that parents donating their child’s tumors unexpectedly found a sense of meaning in their loss. They also found a legacy of their child’s life; the living cells in some ways assisted the parents with grief. Aspects of this sensitive conversation and decision are discussed from the perspective of the parents’ experiences.

Tumor banks play a significant role in allowing scientists to further understand childhood cancer and continue to discover and refine treatments (Balaguer et al., 2006; Oosterhuis et al., 2003). As the treatment for cancer moves ahead, it is imperative to understand the factors which affect research efforts—parental decisions and experiences of tissue donation after death. Unfortunately, rates of tissue donation have declined over the years, presumably due to controversy surrounding ethical and legal issues related to human tissue retention (Seale et al., 2005). It is not well understood what factors affect a parent’s decision to donate his or her child’s tumor postmortem, and what the health care team can do to address parental fears or concerns at a time of great emotional unrest. To the best of our knowledge, this is the first study to address this particular topic about postmortem tumor banking. While much attention has been devoted to autopsy (e.g., collection, reasons for refusal, ethical issues, consent), less is known about tumor banking processes, parental desires, or circumstances around asking for consent. Currently, there exists no “best practice” related to how to ask parents for donation, who should ask and when, what to say, and, as a result, institutions vary in their approach to this. Understanding postmortem tumor banking will lead to refinement to these processes, and ultimately inform what may be considered “best practice” with respect to it. What is unique in this study is the extension of our understanding of the experiences and implications of such decisions and the necessary conversations with families around them. The need for tissue donations is undeniable, but the complexity of how to deliver these conversations well, in a timely way, with tact and sensitivity, acknowledges the impact of both the decision and the delivery of the option of it on families.

## Background

Effective treatment in pediatric cancers is critically dependent on precise histopathological characterization at diagnosis and subsequent assessment of response to therapy (Sebire & Dixon-Woods, 2007). Historically, the mainstay of diagnosis was morphological examination of the tissue via microscope; however, in the last several decades, sophisticated molecular techniques have vastly improved pathologists’ ability to understand and classify tumor types and provide diagnoses leading to specific and effective cancer treatment (Dixon-Woods et al., 2008). These advances in molecular genetics have led to the development of new diagnostic tools, improved prognostic ability, and the development of targeted therapy, thus avoiding toxic therapy for patients whose molecular profile suggests they do not need it (Ferrando & Look, 2004; Khanna & Helman, 2006; Spunt et al., 2012). The future of successful treatment for pediatric oncology patients lies in targeted therapy. The advancement of targeted therapy, still in its infancy, relies on access to tissues or tumors upon which scientists can develop, test, and monitor effectiveness and efficacy of newly created cancer treatments. The way in which tumor samples are most efficiently and reliably obtained is through tumor banks.

Tumor banks are facilities that collect, store, organize, and distribute tumor samples, body fluids, and derivative biomolecules for further use in translational cancer research (Balaguer et al., 2006; Oosterhuis et al., 2003). Specimen quality is of the utmost importance, and tumor banks operate under strict policies and standard operating procedures (Hewitt, 2011). Understanding tumor biology and identifying molecular changes leads to improved risk stratification and treatments (McHale et al., 2007). Appropriate conversations with families, including informed consent discussions, are essential to this process. Many clinical trials require the permission to obtain tissue samples; however, the issue of postmortem donation of tissue and tumor samples is different and much more complex. Postmortem tissue donation has declined over the years, presumably due to controversy surrounding ethical and legal issues related to human tissue retention (Seale et al., 2005). With better understanding around tumor banking, rates could increase again, which could promote better pediatric cancer research and treatment; however, to achieve this, we need to understand the complexity of the issue. The primary purpose in this study was to understand the meaning and impact of tumor donation for parents of children who have died from cancer. We addressed the following research question: How might we understand parental experiences of deciding to donate a deceased child’s tumor to a tumor bank?

## Research Method and Design

This study was conducted using the research method of hermeneutics as articulated by Moules et al. (2015), and as guided by the philosophical hermeneutics of Hans-Georg Gadamer (1960/1989). Guided by philosophy, rather than a conceptual or theoretical framework, hermeneutic research is aimed at understanding rather than explanation. The study was conducted at the Alberta Children’s Hospital (ACH) in collaboration with the Hematology/Oncology/Blood and Marrow Transplant (HOT) program and the Arnie Charbonneau Cancer Institute. Ethics approval was granted from the Health Research Ethics Board of Alberta (Cancer Committee) and institutional approval obtained from Alberta Health Services (HREBA.CC-18-0052). Eligible participants were identified through the tumor bank registry, directed and operated by one of the team members (J. Chan; Clark Smith Pediatric and Neurologic Tumour Bank; Ethics ID REB15_1298). Inclusion criteria for this project were parents of children who have died from a pediatric cancer diagnosis; child’s tumor has been donated no sooner than 6 months from date of this study’s consent; child’s tumor has been donated to a tumor bank; parents speak and read English.

The study team chose to recruit parents whose child’s tumor donation occurred no sooner than 6 months from date of consent to be respectful of the families’ grief, to allow for families to start to adjust to life without their child, and to allow for reflection and insight regarding the tumor banking process.

### Data Collection and Analysis

In Calgary, 19 postmortem tumor banking specimens have been received since 2011 (J. Chan, personal communication, March 25, 2016); therefore, we aimed to recruit five to 10 participants, which is appropriate for a hermeneutic study where the emphasis is on depth and quality, rather than quantity (Moules et al., 2015). Eligible families were contacted via a letter from the office of tumor banking program, and a recruitment poster was posted on the Kids Cancer Care website. We were able to recruit five families and conducted unstructured in person/family interviews with them, the nature of which means that a traditional interview schedule is not used. Demographic information is presented in Table 1; however, because of the small sample size and our desire to protect the identities of the participants as much as possible, only basic information is offered. The interviews were transcribed verbatim and used as the data for the analysis—in other words, the interpretation. Hermeneutics is defined as the tradition, art, and practice of interpretation (Moules et al., 2015); in hermeneutics, data analysis is synonymous with interpretation or understanding. Analysis involved careful reading and rereading of the interviews looking for instances that resonate and that provide portals into understanding. These portals then led to interpretations that are validated by the data themselves in demonstrating a deepening of the understanding of the topic. Hermeneutics is a reflexive and communal endeavor that moves the interpretation between parts and wholes, commonly explained by the metaphor of the hermeneutic circle (Gadamer, 1960/1989; Moules et al., 2015). Hermeneutics does not result in thematic reduction, sematic codes, constructs, or theories (Moules et al., 2015). It aims for an interpretation that extends understanding. As such, the presentation of a hermeneutic study does not follow the tradition of some other qualitative methods; the discussion and interpretation are intertwined with the data and do not stand alone as compartmentalized sections (Moules et al., 2015).

**Table 1.** Demographic Information.

Family	Interviewee	Child’s age at diagnosis	Child’s age at death	Child’s diagnosis
1	Father	2 years	4 years	Hepatoblastoma
2	Mother	Birth (diagnosed in utero)	<1 year	Glioblastoma
3	Father	16 months	9 years	Brainstem glioma
4	Mother	7 years	9 years	Brainstem glioma
5	Mother	17 years	18 years	Rhabdomyosarcoma of face/head

## Interpretive Findings

The findings are presented as interpretations that weave participant voices with the hermeneutic reach that interpretation demands. Participant quotes are presented verbatim and are in italics; bullet points indicate a different participant, and ellipses represent sections of the quote eliminated due to lack of relevance to the particular discussion. As is congruent with hermeneutics, individual participants are not identified. The parents interviewed in this study offered rich and meaningful accounts of their experiences. From their often-painful recollections, we were able to gain a deeper understanding of the complexities of the donation of their child’s tissue and tumors.

### “They’ve Got a Cell Line!”: Living Cells in the Face of Death

Parents facing the death of their child face an unfathomable reality. The death of a child is beyond imaginable and though there are no gradients of grief that can be held up against each other, there are aspects of particular bereavements that are distinctive (Moules, 2009). We expect to bury our parents but not our children. Parents living this experience are living “every parents’ nightmare” but in time they have the capacity to learn that “sadness, joy, memory, celebration can live simultaneously” (Moules, 2009, p. 68). As unimaginable and terrible is this loss, there is hope in it, hope in the belief in our human capacity to suffer, sorrow, heal, celebrate, love, and live. Although it is no consolation, there seems to be something about the continued life of the cells of the child that brought comfort to the parents we interviewed.

*We were sat chatting with her primary nurse and she said to me “they’ve got a cell line. They’ve got a cell line going!”*

*They did get a very enduring cell line that is being researched in another city right now!*

*Received a really good enduring sample . . . and sort of contribute to a future, potentially a future cure, you know.*

*I knew in the meeting before she died that they already had a strong and enduring cell line from previous donations, so in that moment I was able to just kind of let it go. I just said, yes, let’s do it.*

A part of this continued evidence that a piece of their child continues to live, even indefinitely, seems to function to bring a sense of meaning to an experience that really cannot be understood.

### Bringing Meaning

Some of suffering involves a search for meaning and sometimes finding meaning in situations of suffering and loss can work to “soften suffering” (Wright, 2017, p. 3). It cannot and does not eradicate suffering, pain, loss, and grief, but it is a human tendency to try to make sense of things that do not make sense and finding some threads of reason why one’s child had to suffer and then die. In some regards, the decision to donate the child’s tumor/tissue to tumor banking served for some parents as bringing some meaning to the unfathomable situation. Wright et al. (1996) and Wright and Bell (2009) have written extensively about the work of suffering being connected to the act of finding and bringing meaning to situations where meaning seems infinitely elusive.

*The neurosurgeon came in with the paperwork asking if we would consider donating her tumour then to research, and as soon as she said it, it was like, ohhhh, like goosebumps, like oh my God, this is what she’s here for. She’s here to help.*

*Because I kept thinking like why did it take five years for her to come along and why then, and the more and more I thought about it, it was like she was waiting for all the other people who were supposed to be a part of this, assembled and ready in place. And I’m thinking of like Dr. X who works with Dr. Y who works with her cell line and samples, like he worked at Sick Kids before and they recruited him over here and she was waiting for all those puzzle pieces to come together.*

A part of the process of finding meaning in suffering and loss sometimes mirrors parents’ desire to “give back.” In some ways, it could be regarded as a form of altruism; however, altruism is defined as the belief in, or practice of, disinterested and selfless concern for the well-being of others (Altruism, n.d.). Counter to this definition, these parents are not disinterested; they are *very* interested and deeply invested. Their gift is about knowing that the lives of their children mattered and continue to matter in the service of others.

*The fact that it was so rare, this is like gold to researchers, so I couldn’t live the rest of my life knowing that there was this opportunity to donate that could possibly help another family and not consent to do it.*

*Parents really need to understand what benefit could be brought—to help someone else or lead to something . . . if you know it’s used at one university vs. 50 . . . leads to helping many others, at least you could have some solace knowing that.*

*We look for, what can you salvage from a situation, why does a little girl die from cancer? So tumour banking, the way my wife and I both look at it, is an opportunity to take something out of that. So, if she had to do, if this had to happen, I do find comfort knowing that her tumour is contributing to research in a good way. So yeah, had it never been discussed or brought up*, *I would’ve been angry.*

This parent claimed he would have been angry if he had not been given this opportunity to contribute and make his daughter’s life have some meaning and count for something. Finding meaning in loss and the capacity of donation as a way to bring meaning is perhaps one of the primary motivators for parents to donate their beloved child’s tissue. This, however, cannot be upon the suggestion of those initiating this delicate discussion as it is not up to others to decide where meanings lie for different families. In other words, the health care professional having the conversation should not be suggesting that a donation would bring meaning to the parents. Instead, it is the obligation for the health care professionals engaging in the discussion to know how to navigate difficult and sensitive conversations.

### The Conversation: Sensitive Topic, Sensitive Moments

*I guess, given all the conversations around that time, the tumour banking* [conversation] *pales in comparison.*

The conversation around donation of tissues and tumors is necessarily a delicate one that must be handled with the utmost of discretion, sensitivity, timing, and even tact. It requires tact in the way that Gadamer described tact as “. . . a special sensitivity and sensitiveness to situations and how to behave in them, for which knowledge from general principles does not suffice” (Gadamer, 1960/1989, p. 16). This conversation involves this sense of constant tact, employing a knowledge about what to pay attention to, and how to make sense of what is encountered, how to respond to it or not, and knowing when things should be passed over. “But to pass over something does not mean to avert one’s gaze from it, but to keep an eye on it in such a way that rather than knock into it, one slips by it” (Gadamer, 1960/1989, p. 16). This conversation balances how to keep an eye on things that need to be seen and understood, without necessarily addressing those things bluntly or indiscriminately. It involves keeping a focus on something without knocking into it or averting it. This is the attunement that Gadamer so carefully employed in conversation and reminds us of the thoughtful deliberation that donation conversations need to employ in practice. Tact truly is an art of special and knowledgeable sensitivity.

*. . . the most sensitive conversation was held with [CHILD]’s oncology doctor, you know, the doctor I know well. So, I guess from that perspective, it, the most sensitive conversation happened with the right people I guess you could say. So I think the more sensitive circumstances, the more important it is that the people that the parents know and are comfortable with are actually in the room having that conversation. I guess my only advice is the higher the degree of sensitivity, the more important it is that trusted people be there. And I would say if you, if there is a sense that the parents are reluctant, I’d say it’s even more important that the person that they have the best rapport with be part of it.*

*. . . it all comes down to sensitivity and really having an understanding of the parents and how they’re going to potentially react to things Dr. [NAME] did approach me very quietly and said, “can we take a few moments to go and discuss this”? He introduced me to everybody, we have a room over here, let’s just go sit down. But you are a little bit bewildered and yeah. The request to have the conversation was again, somebody that I knew, that I trusted, quiet, you know, a private thing, and we’re going to meet with these other two people and I understood why they needed to be there, and that sort of thing. If I had thought that it wasn’t handled for the sensitivity of the moment, not even the sensitivity of the asking for the tumour to be donated, but for the sensitivity of the moment, you know. I do think that the proper mood, for what was going on in the other room, I think that was respected. And that is very important.*

*. . . the discussion, when to have the discussion about tumour banking is a hard one to research I think, because it’s so personal. It depends so much on the point of view. And it comes down to the doctor’s understanding of the parents. By that point, you know, we had been dealing with [CHILD]’s doctor for two years. So you have a bit of a relationship, a bit of a rapport. He’s sat down in front of us and given us bad news, unfortunately, many times. Ultimately, it’s a personal decision and no amount of research will answer it definitively.*

*My overall, I guess I feel like I’ve given my overall impressions about how it was handled, and coming away with those, at the end of the day, how the question was worded or what is the . . . the emotion behind it was respected.*

A part of tact is the attention to timing. Knowing when to address things is as important as knowing what to address and how to do it.

### Timing: A Fine Balance

Parents told us they did not remember much of what they were told. Postmortem tumor banking is not just the act of taking the tumor and banking it. It starts with the first conversation and in many ways never ends, but there is a judgment about timing about the conversation and what is offered at particular times.

*I don’t know, I’m assuming [TUMOR BANKING DOCTOR] told us when she came in to meet with us, but I can’t remember. And I know she gave us quite a good overview but I honestly don’t remember a lot of what she said.*

*Because obviously as things sink in more, more questions come about so we would be asking different things, and they were always very good at explaining things.*

There is also timing around the grief process. Parents who know they are going to lose their child are thrust into individual experiences of anticipatory grief, a term first coined by Lindemann in 1944 (Lindemann, 1944/2006). His characterization of it was a reaction to the threat of death rather than the actual death, whereas Rando (2000) felt that grief was only one component of the experience of coping, interaction, planning, and psychosocial shifting. Alternately, she offered the term anticipatory mourning which encompasses all of these processes. Raffin Bouchal et al. (2015) furthered this idea in suggesting that it is a process of holding on and letting go. We offer that the parents who know their child is dying are immersed in a process of looking forward, looking backwards, and living in-between, as they think to a past with their child, anticipate a future without their child, and live in the middle of both realities.

*They took us over to the Rotary House and we just met the staff, we met our grief counsellor, but I left that day thinking, well that’s awfully nice but these people don’t understand that, you know, something’s going to happen, and she’s going to be okay. You know? So I think the denial, so I don’t know, you know, I still think we would’ve reacted okay to that conversation a little bit sooner, but from the perspective of the doctors, I think it was pretty clear that we were in denial about where we were at with her life.*

Perhaps in the face of the many conversations that happen in this time, the tumor banking pales in comparison. Perhaps the conversations have to be linked such as in having palliative care conversation and the banking conversation together. Perhaps if the groundwork is laid, the conversation can evolve as naturally as a conversation can evolve in such circumstances.

*I do think it was, you know, brought up, even just as a high-level thing. Like at periods throughout this journey, you may be asked to have a conversation about tumour banking or that sort of thing. I do think that we got some forewarning, again, maybe the conversation about tumour banking at the time of her death, I think it might have been better to have that conversation, actually around the time that we had the, when we discussed do not resuscitate.*

*I guess the most sensitive issue was timing because again, the sooner after surgery or death, the better to get the sample with the chances of keeping it alive and enduring. So, they, I guess that was the hardest part of the conversation, when they said that, ideally it would be within two hours after she passed away. And, like in that situation I like I have no idea what those moments will be like, and I gave them, I guess permission. I said*, “*look, I won’t be upset if you prompt us, like just a gentle reminder, like say an hour, an hour and a half, two hours, and we can maybe make a decision then.*”

*And emotions change too, right? Like from each phase, it sounds like it was more hopeful.*

There is no good time for this conversation, a conversation that no parent wants to have. Still, timing matters.

*It was a big step for us, emotionally not cognitively, we kind of just sat down, like they gave us, they said take as much time as you need, we can cover this tomorrow or if you want to take some time to think about it, cognitively we understood. We’re smart people, we know what’s going on. It was just that emotional step of saying the words out loud*: “*we’ll let her go.*” *So that’s all very intense, we got through that, I would think that within maybe a day or two of that conversation would be an okay time to, give you some time to calm down from that, and then, and that’s probably okay to talk about tumour donation at that time. And even explain, you know, like there’s really no good time to have this conversation, but let’s talk about it now because in her final moments, we don’t want to be having this conversation. because in a way they’re not only asking you about tumour banking, they’re just sort of confirming that your child’s going to die.*

### Who to Hear From and What to Hear

The knowledge of who the doctor was, who would remove the tumor, and where it would be banked seemed to personalize it or make it less unknown and, in one sense, more palatable and comforting. Perhaps this is akin to the act of picturing the monster and how it seems to diminish the power of it and take some of the fear out of it. We all fear things that are frightening and discussing or even considering the death of your child and subsequent isolation of tissue from your child can become a monster in thought (Lankton & Lankton, 1989, pp. 106–110). However, opening the door to the stranger (personalization of the strangeness of the situation) takes a risk that the stranger might be a monster or a messiah (Kearney, 2004). There is something monstrous to imagine your child’s tissue but the parents in this study showed us that there was a gift in making this decision that helped to soften their suffering and give some meaning to their child’s death. There is also something that diminishes the strangeness by meeting the doctor and entrusting the doctor to take care of a part of their child, a part that they can no longer care for.

*So for me to actually meet the doctor who was going to do the removal . . . just to have that face, the knowledge of the process . . .*

*I remember looking at (the doctor), and she’s such a smiley person, she’s lovely, and I just remember looking at her and thinking, god, you’ve got such a good way about you and we’re talking about this horrible thing and I just loved her smile. I picked up on her enthusiasm for her job for what she did, her passion. You could see how dedicated she was. And I was* *thinking knowing (the child)’s legacy was going to be in her hands; she’s now looking after my little girl as such. I knew she was in really good hands.*

What was discussed was also important; there are things parents want to know and things they do not want to know.

*I did tell them, I don’t know if it’s because of the way the ethics work, I did say, “if you’re going to take it, I’d like you to get a good sample, but I don’t necessarily want to know what you had to do to get it,” right?*

*Don’t do anything invasive if you don’t need to, but if you do, it’s okay, but . . . just don’t tell us about it.*

*The concern for me was I didn’t want to see her afterwards and for her to look a mess. I didn’t want that to be my last image of [CHILD]. I said, “will they go in where her existing scar is where they’ve done the surgeries before”? It wasn’t going to change my mind about doing it but I needed her to be dressed in a way that I won’t see that.*

*He (the father) wanted them to take everything, the whole tumour, everything that they could get out. Because he wanted her to finally be free of cancer.*

Reading the family’s cues around all aspects of the conversation—timing, location, who, and what—was noticed by the parents. What kinds of information to be shared differed for each family. The kinds of reassurances needed were not even necessarily known by the parents. How does anyone know in these kinds of moments what they can hear and not hear, or what they want to know and not know? The discernment necessary for this again returns to the sensitivity of the conversation and the tactful questioning and reading of the parents at the time.

### Transport: The Longest and Shortest Hallway

The conversation of considering donation of tissue does not end with the consent being signed. There is a process involved. A part of the process can involve the transport of the child to the pathology lab.

*And I remember her nurses just going into so much detail about what happens on the unit in terms of why the doors are all shut off. So, like where we were, as we would come out of our room, she says so these main doors, they’ll close them so nobody on the unit sees anything. And that we be taking her right through the back area . . . it was up on the third floor. Which I didn’t like actually. I didn’t like having to walk through the corridors because although you go through the back area where the elevators are that the porters use, you then go up onto the main corridor. Thankfully it was a Sunday, so the hospital was quiet and we only passed by one person while we were going to the pathology suite. And because [CHILD] was a baby and wrapped and I was carrying her, they wouldn’t have known. But I thought for some families whose child has just died who are just devastated doing this walk. I wanted to be with [CHILD] as much as possible. So we walked her there and with the security guard that although they are slightly ahead, other people might figure that they’re with you but if it had been a busier day and those corridors were busier, that might have been more difficult.*

Parents in the hospital where we interviewed are given the option of accompanying their child to the pathology lab. It seemed important that this be given as an option. For those that took it, the walk down the long hospital corridors and rides in the back elevators, spending last precious moments with their child’s body was a walk of intense intimacy open to public display. The carefulness of the health care providers who prepared the parents for this walk was noticed and deeply appreciated.

### Grief and Legacies

*If we have to have these conversations, let’s have them. Check it all off and I can get on with the business of grieving.*

In the end, this study shows us that the topic of postmortem tumor banking is intimately tied to grief. It is obvious that parents whose child had died are acutely grieving but what emerged in this study was the connection that tumor banking had to grief and how it played a role in offering some comfort to the parents in their grief. It not only brought meaning to the terrible loss they are enduring but it also serves as a way for the parents to stay connected to their child. It brings a living legacy to their child’s life.

*She showed us her cells. We looked at them under a microscope. That was amazing. To actually see a part of her legacy . . . so much comfort for me. Walking around* (the laboratory) *taking photos of everything, like, I’m so proud of my daughter’s place of work.*

This participant informed us that she emailed the researcher regularly to check on her daughter’s cell line, as though a connection to this legacy was a part of her grieving process. Grief is very much about maintaining connection to the deceased. When asked by the interviewer if the decision to tumor bank helped in any way for the parent to stay connected to her child, she responded,

*To know that she’s, that I do still have that connection at the moment, it’s a bonus. It didn’t form my decision, initially, because at that time I didn’t know that we would still hear about what was going on.*

Older conceptualizations of grief characterized it as occurring in stages that eventually involved successful separation between the living and the deceased and a resolution of feelings of grief as measured by the absence of such feelings. However, newer conceptualizations have emerged that more accurately reflect what really happens in grief. Grief is not a staged, time-dependent process; it is a living, ongoing, lifelong experience that changes in nature over time and rather than disengaging with the deceased, it is about the living finding a new relationship and connection to the one they have lost (Moules, 1998; Neimeyer, 2001a, 2001b). Rather than letting go, the loss and the continuing bond is negotiated and renegotiated over time (Klass et al., 1996; Moules et al., 2004; Neimeyer, 2001a, 2001b, 2016; Neimeyer et al., 2006). Neimeyer (2016) described grief as a process of affirming or reconstructing personal meaning into a world that has been transformed by loss.

George (2017) reminds us that a part of grieving is the “ethical demand to carry the memory of the deceased into the future” (p. 5). The legacy of living tissue of one’s loved child is one way that child is carried into the future. Citing a line from Paul Celan’s poetry, he quotes Derrida (2005), “The world is gone, I must carry you” (p. 141).

While our friend or loved one is still alive, our relation is . . . carried out by us both, mutually, and in the flesh. Once our friend or loved one is dead, however, our relations can no longer be carried out by us both in the flesh but must rather be carried on only by the survivor and only in memory. If grieving involves us in the limit situation of memory, and indeed, a limit that makes of grieving an infinite task, then this entails the ethical demand to carry the memory of the dead. (George, 2017, pp. 4–5)

The living legacy of a deceased child in no sense compensates for the infinite task of grieving these parents experience, but perhaps there is some degree of knowing they are carrying their child that might offer some comfort and solace.

## Implications for Practice

The parents in this study offered some suggestions that might serve as recommendations for health care professionals in working with families around the issue of tumor banking. One involved the process of actual removal of the tumor or tissue.

*There’s a little room at the side of the pathology suite . . . And it’s a really lovely little waiting room but there’s a connecting door I think must open to the pathology suite because I could hear people moving things about, and putting things down, and it sounded like, you know steel and what not. And I remember sitting there thinking, “Oh my God, are we supposed to sit here? And I’m going to hear what they’re doing?” And as much as I wanted to be a part of [CHILD] and I was Googling her operations and things before, I didn’t want to know anything more about that bit. And when they came and took [CHILD] I think the social worker said to us, do you want to stay here or do you want to go back to the unit? And I was like, I’m going back to the unit. I think that’s a good head’s up for parents because I can’t even imagine what trauma that leaves people. And once you’ve heard something fire up and start cutting away, because particularly in [CHILD’s] case, they’ve got to cut through bone, and it’s not just cutting skin, and that would’ve been horrific to hear.*

Other hospitals may be designed differently but what is suggested here is that, although parents are encouraged to remain with their child for as long as they desire, there might be a point for some parents that being present might create more pain. An awareness of this is important.

There was something about having an orientation to the setting that mattered to some of the parents, both the physical location as well as to the people involved.

*Maybe a tour of the lab ahead of time to see what is involved and see some of the doctors.*

The amount of information and the timing of it prompted the suggestion of having written material, as well as the opportunity to revisit as questions arose.

*But again, it’s all so hazy in that moment because you’re trying to process so much, so I think having something written down, just very simple and straight forward so that it kind of gives you a heads up on what to expect, not medical wise, but the whole process of getting her from the room to there and about this window of opportunity . . . just because you’re taking so much in, it’s good to have that to kind of refer back to, as questions come in and that prompts questions and conversations as well.*

Some kind of follow-up after the donation was important to some of the parents, knowing the legacy of their child was continuing, if the cell line was growing, and ultimately if it was contributing to any kind of significant discovery.

*Letting us know if there is something significant . . . if it leads to something. It might help with the grieving process—to find out if something good happened or any discovered.*

In line with this, one parent suggested that even a general newsletter about all the discoveries in the lab would be appreciated, perhaps even generally to know their child was a part of the progress being made.

*Maybe a newsletter from the lab what new discoveries they are making.*

A very concrete but useful suggestion was made about offering families a tangible acknowledgment of the contribution the child has made in the form of a certificate. The idea of having such a document might perhaps serve as a reminder of the generosity and legacy.

*Maybe a certificate stating it was something our child did for future generations.*

In general, the parents in this study did not have criticisms of how each of their requests, conversations, and donations occurred, but they remind us of best practices in navigating sensitive conversations with tact, timing, the right information, and the right people.

## Conclusion: Enduring Cell Lines; Enduring Love

Once thought of as a death sentence, childhood cancer is one of the undisputed success stories of modern medicine. Improvement in survival rates can be attributed to refined diagnostic procedures, multimodal therapies, centralization of care and support services, high levels of participation in clinical trials, and participation in investigations that use tissue postmortem (American Cancer Society, 2015; Canadian Cancer Society, 2020; Siegel et al., 2012). Despite significant improvement in survival rates and understanding about pediatric cancers, childhood cancer remains the second leading cause of death in children aged 1 to 14 years (Canadian Cancer Society, 2020); there is much to learn. Tumor banks play a significant role in allowing scientists to further understand this disease and continue to discover and refine treatments.

This study has helped us to understand what it means to the parents of children needing to make this decision about whether to donate the tissue of their child after death. It has given insight in how we might host these families and their grief with grace and sensitivity. Losing a child is unimaginable and making the decision to allow one’s child’s tissue to continue life while the child no longer lives is a complex and unfathomable experience. As parents are faced with the death of their child, finding the strength and capacity to make this decision is not only courageous, but also generous and loving. The capacity to learn of the enduring cell line of one’s child and the potential for it to make a difference in the lives of other children is at once filled with one kind of answer to an unanswerable question, as well as filled with the deep well of grief that, though the cells endure, one’s child is no longer alive. As health professionals, we need to learn how best to facilitate and guide these unfathomable conversations and honor and thank the parents who are able to walk that long hallway.
